# Fusion of DNA-binding domain of *Pyrococcus furiosus* ligase with *Taq*Stoffel DNA polymerase as a useful tool in PCR with difficult targets

**DOI:** 10.1007/s00253-017-8560-6

**Published:** 2017-11-04

**Authors:** Marta Śpibida, Beata Krawczyk, Beata Zalewska-Piątek, Rafał Piątek, Magdalena Wysocka, Marcin Olszewski

**Affiliations:** Department of Molecular Biotechnology and Microbiology, Gdańsk University of Technology, ul. G. Narutowicza 11/12, 80-233 Gdańsk, Poland

**Keywords:** PCR, DNA polymerase, Fusion DNA polymerases, Archaeon

## Abstract

The DNA coding sequence of *Taq*Stoffel polymerase was fused with the DNA-binding domain of *Pyrococcus furiosus* ligase. The resulting novel recombinant gene was cloned and expressed in *E. coli*. The recombinant enzyme was purified and its enzymatic features were studied. The fusion protein (*Pfu*DBDlig-*Taq*S) was found to have enhanced processivity as a result of the conversion of the *Taq*DNA polymerase from a relatively low processive to a highly processive enzyme. The abovementioned processivity enhancement was about threefold as compared to the recombinant *Taq*Stoffel DNA polymerase (*Taq*S), and the recombinant fusion protein was more thermostable. It had a half-life of 23 min at 99 °C as compared to 10 min for *Taq*S. The fusion protein also showed a significantly higher resistance to PCR inhibitors such as heparin or lactoferrin and the fusion polymerase-amplified GC-rich templates much more efficiently and was efficient even with 78% GC pairs.

## Introduction

At the core of PCR-based techniques is the use of a DNA polymerase which plays the major role in molecular biological applications including DNA amplification or sequencing (Terpe 2013). PCR requires a high temperature, and therefore, it is necessary to use a thermostable enzyme for DNA amplification; the most commonly used enzyme is a DNA polymerase derived from the thermophilic *Thermus aquaticus*. Its native form is produced in *Thermus aquaticus* and its recombinant form by *Escherichia coli*. The *Taq*’*s* half-life is short compared to that of the other polymerases, e.g., these isolated from Archaea: only 40 min at 95 °C or 9 min at 97.5 °C (Lawyer et al. 1993). Furthermore, the *Taq* DNA polymerase is not able to synthesize products which are longer than 4 kb (Hamilton et al. 2001). In general, *Taq* amplification efficiencies for targets shorter than 1 kb, with 45 to 56% CG content, lie at about 80% (Arezi et al. 2003). Usually, product yields decrease when the amplicon size becomes greater than 1 kb because of a relatively low processivity and thermostability of a wild-type enzyme. A novel strategy used to enhance the processivity and performance of the *Taq* is to fuse it with a thermostable DNA-binding protein such as a Sso7d DNA-binding protein derived from *Sulfolobus solfataricus* (Wang et al. 2004).

We have tried to improve the properties of the *Taq* polymerase by creating a fusion protein which contained a highly thermostable DNA-binding domain of the *Pyrococcus furiosus* ligase, a 6-amino acid linker and a sequence of the *Taq*Stoffel polymerase.

## Materials and methods

### Construction of recombinant plasmids

The *T. aquaticus* strain (ATCC25104) was used to isolate a genomic DNA which was used as a template for the amplification of a *taq*Stoffel fragment gene with the use of a standard PCR amplification protocol with the Marathon DNA polymerase (A&A Biotechnology, Gdansk, Poland). A DNA fragment of *taq*Stoffel corresponding to nucleotides 997 to 2626 was obtained in PCR with the use of primers: 5’*AATTTTGTTTAACTTTAAGAAGGAGATATA*CATATGGCCCTGGAGGAGGCCC

(forward) and 5′ *GCAAGCTTGTCGACGGAGCTCGAATTC*GGATCCTTAatggtggtggtggtggtgCTCCTTGGCGGAGAGCCAG (reverse). The primers contained sequences complementary to the *taq*Stoffel gene (underlined), a sequence complementary to the pET-30 Ek/LIC vector (italics), and a sequence for the oligohistidine tag (lowercase). The stop codon (TTA) was added to the reverse primer immediately after the oligohistidine sequence.

The DNA-binding domain of *Pyrococcus furiosus* ligase gene was fused with a DNA fragment of *taq*Stoffel (corresponding to nucleotides 997 to 2626 of *taq* gene) in PCR using primers: 5’TATTGGCTTTC**GGAAGCGGAGGGGTCGAC** GCCCTGGAGGAGGCCC (forward) and 5′ *GCAAGCTTGTCGACGGAGCTCGAATTC*GGATCCTTAatggtggtggtggtggtgCTCCTTGGCGGAGAGCCAG (reverse). The primers contained sequences complementary to the *taq*Stoffel gene (underlined), a sequence complementary to the pET-30 Ek/LIC vector (italics), a sequence for a 6-amino acid residue linker (bolded), and a sequence for the oligohistidine tag (lowercase). The stop codon (TTA) was added to the reverse primer immediately after the oligohistidine sequence.

The genomic DNA of *Pyrococcus furiosus* (DSM 3638) was used as a template for the amplification of the DNA-binding domain of the ligase gene (accession number: NC_003413) using a standard PCR amplification protocol. The forward primer was 5′ *ATTTTGTTTAACTTTAAGAAGGAGATATA*CATATGCCATTGTGGGCTTTATCTTCAACCA (containing a sequence complementary to the DBD fragment of the ligase gene (underlined), and a sequence complementary to the pET-30 Ek/LIC vector (italics) and the reverse primer was 5′.

TCCTCCAGGGC**GTCGACCCCTCCGCTTCC** GAAAGCCAATAAAGCCAATGCTTGC (containing a sequence complementary to the DBD fragment of the ligase gene (underlined) and a sequence for a 6-amino acid residue linker (bolded). As a result of the PCR amplification, the following two products were obtained: the *taq*Stoffel gene (1703 bp) and the DBD fragment of the ligase gene (685 bp). The PCR products were then mixed together with the DNA of the pET-30 Ek/LIC vector (Novagen, Madison, WI, USA) digested with the use of *Bam*HI and *Nde*I enzymes (NEB, UK), and the mixture was used for cloning with the use of the OverLap Assembly kit (A&A Biotechnology, Poland). The resulting pET30/PfuDBDlig-TaqS plasmid had a reading frame which contained the DBD sequence of *P. furiosus* ligase (amino acid residues 218 to 424), a 6-amino acid linker GSGGVD), a sequence of the *Taq*Stoffel polymerase (amino acid residues 317 to 832), and a His tag domain which was necessary for the purification of the recombinant protein by metal affinity chromatography.

The nucleotide sequences of the resulting recombinant plasmids, pET30/TaqS and pET30/PfuDBDlig-TaqS, were confirmed by DNA sequencing (Genomed, Poland).

### Expression and purification of *Taq*S and *Pfu*DBDlig-*Taq*S polymerases

The pET30/TaqS and pET30/PfuDBDlig-TaqS plasmids were transformed into *E. coli* BL21 (DE3) RIL (Novagen, USA). The cells which carried the required plasmids were grown at 37 °C in the Luria-Bertani medium, supplemented with 50 μg/ml of kanamycin and 50 μg/ml of chloramphenicol to an OD 600 of 0.4 and were induced by incubation in the presence of IPTG, at a final concentration of 1 mM, for 24 h. The cells were then harvested by centrifugation and the pellets were resuspended in 20 ml of buffer A (50 mM Tris–HCl pH 9, 0.5 M NaCl and 5 mM imidazole). The samples were sonicated three times for 30 s at 4 °C and centrifuged for 15 min at 10000×*g*. The supernatant was heat-treated for 30 min at 70 °C, and the denatured host proteins were removed by centrifugation. The protein was next purified in a one-step procedure using Ni^2+^-affinity chromatography. The supernatant containing the protein was loaded directly into a His•Bind Column (Novagen, USA) prepared and equilibrated using the A buffer. The recombinant proteins were washed twice with the washing buffer B (50 mM Tris–HCl pH 9, 0.5 M NaCl and 40 mM imidazole) and then eluted with the elution buffer E (50 mM Tris–HCl pH 9, 0.5 M NaCl and 300 mM imidazole). The eluted fractions were dialyzed three times against the buffer D (100 mM Tris–HCl pH 8, 100 mM KCl, 0.2 mM EDTA). To remove the trace amounts of the genomic bacterial DNA, 25 U of Benzonase® endonuclease (Merck, Darmstadt, Germany) and MgCl_2_ were added at the final concentration of 5 mM and the preparation was incubated for 1 h at 37 °C. Next, Benzonase was inactivated by incubation for 15 min at 70 °C, and following this, the denatured proteins were removed by centrifugation. Finally, the preparations were dialyzed once against the storage buffer (50 mM Tris–HCl pH 8, 50 mM KCl, 1 mM DTT, 0.1 mM EDTA, 1% Tween 20, 1% Nonidet P-40 and 50% glycerol).

### DNA polymerase activity assay

The activity of the DNA polymerase was measured in an isothermal reaction at 72 °C using a real-time PCR apparatus (IT-IS International Ltd., UK) and EvaEZFluorometric Polymerase Activity Assay Kit (Biotium, Hayward, USA) in accordance with the definition of one unit of enzyme activity by Tveit and Kristensen (“One unit of DNA polymerase activity is conventionally defined as the amount of enzyme that will incorporate 10 nmol of nucleotides during a 30-min incubation”). When the DNA polymerase is active, the primer is extended to form a double-stranded product which binds the EvaGreen dye, with the resulting increase in fluorescence. The rate of increase shows a positive correlation with the polymerase activity (Tveit and Kristensen 2001; Driscoll et al. 2014; Ma et al. 2006). The activity was determined using the commercial *Taq* polymerase (Thermo Scientific, USA) with an activity of 1 U/μl as a reference.

### Optimization of PCR amplification

To optimize the amplification process, the polymerase activity was measured using various concentrations of MgCl_2_, KCl and (NH_4_)_2_SO_4_ in the buffer and various pHs. All reactions were performed using 1 mM of each dNTP, 0.4 mM of each primer and, as a template for PCR, the pET 30 plasmid DNA containing a known target sequence (PCR product of 300 bp). The PCR experiment was performed using 1 U of purified *Pfu*DBDlig-*Taq*S DNA or *Taq*S DNA polymerase in 20 μl of the reaction mixture containing 50 ng of the DNA template. The PCR experiment was conducted as follows: 1 min at 94 °C; 25 cycles of 15 s at 94, 55, and 72 °C. To determine the optimum MgCl_2_ concentration, the PCR was performed at increasing concentrations of MgCl_2_ (0–9 mM) with the use of the Tris–HCl buffer. Furthermore, the PCR experiment was carried out using various concentrations of KCl (10–90 mM) and (NH_4_)_2_SO_4_ (10–90 mM), at various pHs (7.0–9.0), with the use of the Tris–HCl buffer.

Temperature stability was assayed as described by Dabrowski and Kur (1998). One unit of purified *Pfu*DBDlig-*Taq*S and *Taq*S DNA polymerases were heated at 95 and 99 °C for 1, 5, 10, 20, 40, and 60 min. The same amount of enzyme was used to amplify a 300-bp target fragment under the optimal reaction conditions (in the same PCR experiment and under the conditions as determined in the optimization process): 20 mM Tris–HCl (pH 8.0), 4 mM MgCl_2_, 10 mM (NH_4_)_2_SO_4_, and 10 mM KCl.

### Measurements of PCR amplification rates

The PCR amplification rates were measured using the method described by Lee et al. 2010, after some modifications. *Pfu*DBDlig-*Taq*S and *Taq*SDNA polymerases were used to amplify the PCR products under the same conditions as those determined in the optimization process and with the use, as a template for PCR, of the pET 30 plasmid DNA containing a known target sequence (PCR product of 300, 500, and 1000 bp). Each PCR cycle started with an initial denaturation at 94 °C for 2 min and included 25 cycles of 15 s at 94 and 55 °C and 25 cycles of 5 to 60 s at 72 °C.

### Processivity

The processivity test was carried out as described by Elshawadfy et al. 2014, with some modifications. Eighty-five microliters of reaction buffer (20 mM Tris–HCl pH 8.3, 10 mM KCl, 10 mM (NH_4_)_2_SO_4_, 0.1% Triton X-100), 290 μM of each of the four dNTPs, 40 nM primer template (5′-GGGGATCCTCTAGAGTCGACCTGC and 5′ TATCGGTCCATGAGACAAGCTTGCTTGCCAGCAGGTCGACTCTAGAGGATCCCC), 3 μl of EvaGreen Fluorescent DNA stain (Jena Bioscience, Jena, Germany), and 1 U of tested polymerase were pre-incubated at 50 °C for 5 min. Reactions were initiated by the simultaneous addition of 7.5 μl of 50 mM MgCl_2_ and 7.5 μl of 0.6 mg/μl of a heparin trap, and the polymerization process was allowed to proceed at 72 °C. Aliquots of 10 μl were withdrawn to cold tubes (4 °C) at 0 (before polymerization process was allowed), 1, 2, 5, and 10 min after polymerization process was allowed and the extension was determined by melting the obtained products with the use of a real-time PCR MyGo instrument (IT-IS International Ltd., GB). The reaction included a pre-melt hold at 95 °C for 10 s (a ramp rate of 5 °C/s), an initial stage at 60 °C for 60 s (a ramp rate of 4 °C/s) and the final stage at 97 °C for 1 s (a ramp rate 0.201 °C/s). The processivity was determined by comparing the melting temperature profiles of the obtained products with the profiles of products with a known length, serving as markers in the reaction. Marker products were obtained in reactions which used the same primer and synthetic templates, which allowed the formation of products which were longer than the primer by 1, 2, 3…22 nt.

### Sensitivity

Polymerase sensitivity (affinity for the template) was measured using a PCR in accordance with the protocol described by Halley and Prezioso (2003), after some modifications. The PCR was conducted under conditions optimized for the fusion polymerases *Pfu*DBDlig-*Taq*S and *Taq*S. Plasmid DNA pET28a(+) (Novagen, Madison, USA) was used as a template along with the primers GATGCTGCTGGCTACCCTG (forward) and TCAAGAACTCTGTAGCACCGC (reverse), and the product of the reaction was 1264 bp. The reaction was conducted at decreasing template concentrations (serial two-fold dilutions of template) and the reaction included an initial denaturation at 94 °C for 2 min followed by 25 cycles of 15 s at 94 and 55 °C and of 60 s at 72 °C. The amplified products were analyzed in a 1.2 % agarose gel stained with ethidium bromide.

### Resistance to inhibitors

The effect of PCR inhibitors such as lactoferrin (Sigma-Aldrich, St. Louis, USA) in a range from 54 to 0.84 μg, heparin (Sigma-Aldrich, St. Louis, USA) in a range from 600 to 4.7 ng, and human blood (from a healthy individual) in a range from 10 to 0.15%, on the catalytic activity of the *Pfu*DBDlig-*Taq*S and *Taq*S DNA polymerases, was assessed by a PCR reaction using the genomic DNA of *Staphylococcus aureus* as a template and primers for the specific *nuc* gene detection as described by Barski et al. 1996.

### Efficiency of a long-range PCR

The efficiency of a long-range PCR was measured as described by Kwona et al. 2016, after some modifications. The PCR experiment was performed with the use of 1 U of purified *Pfu*DBDlig-*Taq*S DNA polymerase and also with the use of *Taq*S DNA polymerase which were placed in a 20 μl reaction mixture containing 50 ng of the *E. coli* genomic DNA as a template. Primers were used to amplify the following four DNA fragments: 5 kbp (5′ GCACCATCAACAATAAAGGCGC and 5′ TTCCGCTAATGCCATGGTGATAG), 8 kbp (5′ GCACCATCAACAATAAAGGCGC and 5′ AACGATGCGATATAGCCGACAC), and 10 kbp (5′ GCACCATCAACAATAAAGGCGC and 5′ AACGATGCGATATAGCCGACAC). The PCR experiment was conducted as follows: 2 min at 94 °C; 30 cycles of 30 s at 94 and 56 °C, and 60 s/kb at 72 °C. The amplified products were analyzed in a 1% agarose gel stained with ethidium bromide.

### GC-rich templates

The efficiency of the amplification of the products which are rich in GC pairs was evaluated using a GC-rich template of *Mycobacterium tuberculosis.* The use of the forward primer CCGCCGTTACCACCCTTACCACCGTT and the reverse primer GCACCGCACCCACCAGCGGC enabled the production of a target with a length of 301 bp and with a GC content of 78% (Kotłowski 2015). The reaction took place under the conditions which were optimized for the fusion polymerase *Pfu*DBDlig-*Taq*S and reference *Taq*S at an increasing polymerase activity. It started with 3 min at 95 °C followed by 35 cycles of 15 s at 94, 64 and 72 °C. The amplified products were analyzed in a 2% agarose gel stained with ethidium bromide.

## Results

### Expression and purification of *Taq*S and *Pfu*DBDlig-*Taq*S polymerases

**Fig. 1:**
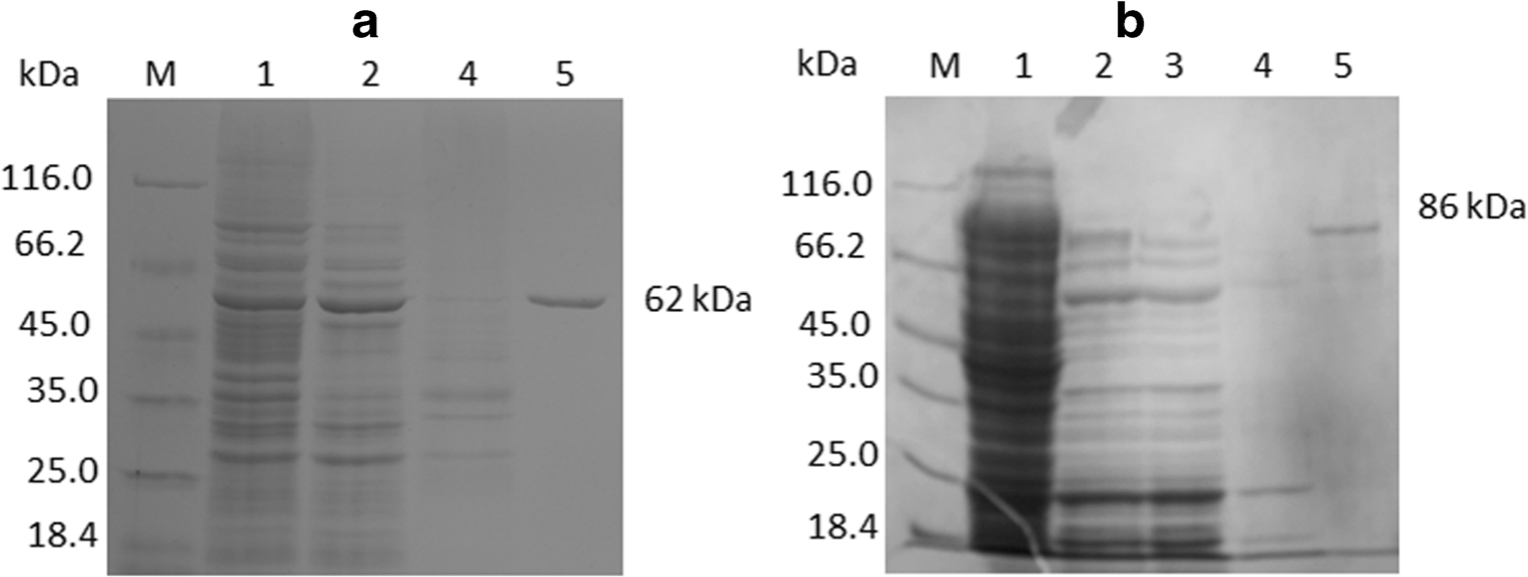
Expression and purification of the TaqS (a) and the PfuDBDlig-TaqS (b) polymerases. The proteins were analyzed on a 10% polyacrylamide gel (SDS–PAGE). Lane M, unstained protein weight marker 26.610 (14.4–116) (ThermoScientific, USA), with the molecular mass of proteins marked. Lane 1, sonicated extract of induced cells; lane 2, heat treatment; lane 3, fraction of unbound proteins; lane 4, fraction after second washing with buffer B; lane 5, purified protein after elution with buffer E

The gene encoding a Stoffel fragment of the *Taq* polymerase was cloned into a pET-30 Ek/LIC vector to generate a pET30/TaqS plasmid, leading to the expression of the enzyme as a fusion protein with a C-terminal polyhistidine tag. To achieve fusion with the DNA-binding domain of *Pyrococcus furiosus* ligase gene, two PCR products were mixed together with the DNA of the pET-30 Ek/LIC vector DNA to generate the pET30/PfuDBDlig-TaqS plasmid coding the fusion enzyme with a C-terminal polyhistidine tag. *E. coli* BL21 (DE3) RIL cultures harboring recombinant plasmids were generated, and cells were harvested and sonicated. The recombinant DNA polymerases were purified by passing the heat-denatured supernatant through a His•BindNi^2+^ affinity column. The specific activities of the purified *Taq*S and *Pfu*DBDlig-*Taq*S polymerases were found to be 1800 and 1600 U/mg, respectively. These results indicated that the *Pfu*DBDlig fusion did not have a negative effect on the catalytic activity of the *Taq*S DNA polymerase. After each purification step, the purity of the DNA polymerase was monitored by SDS–polyacrylamide gel electrophoresis (Fig. 1) which separated the following major protein bands: 62 or 86 kDa for *Taq*S and *Pfu*DBDlig-*Taq*S, respectively; this result was in agreement with the molecular masses of 61.8 or 86.1 kDa which were calculated based on the amino acid sequences. The *E. coli* overexpression system used in this study enabled the production of 30 mg of *Taq*S polymerase and 20 mg of *Pfu*DBDlig-*Taq*S fusion protein per 1 l of induced culture. The production efficiency of the *Taq*S and *Pfu*DBDlig-*Taq*S polymerase in this study was quite satisfactory.

### Characterization of the fusion *Pfu*DBDlig-*Taq*S DNA polymerase

In our experiments, the activity of 1 U/μl was determined for *Taq*S and *Pfu*DBDlig-*Taq*S DNA polymerases and was compared to the commercial *Taq* polymerase with an activity of 1 U/μl, using the EvaEZFluorometric Polymerase Activity Assay Kit (Biotium, Hayward, USA) in an isothermal reaction at 72 °C on a real-time PCR apparatus (IT-IS International Ltd., UK).

**Fig. 2:**
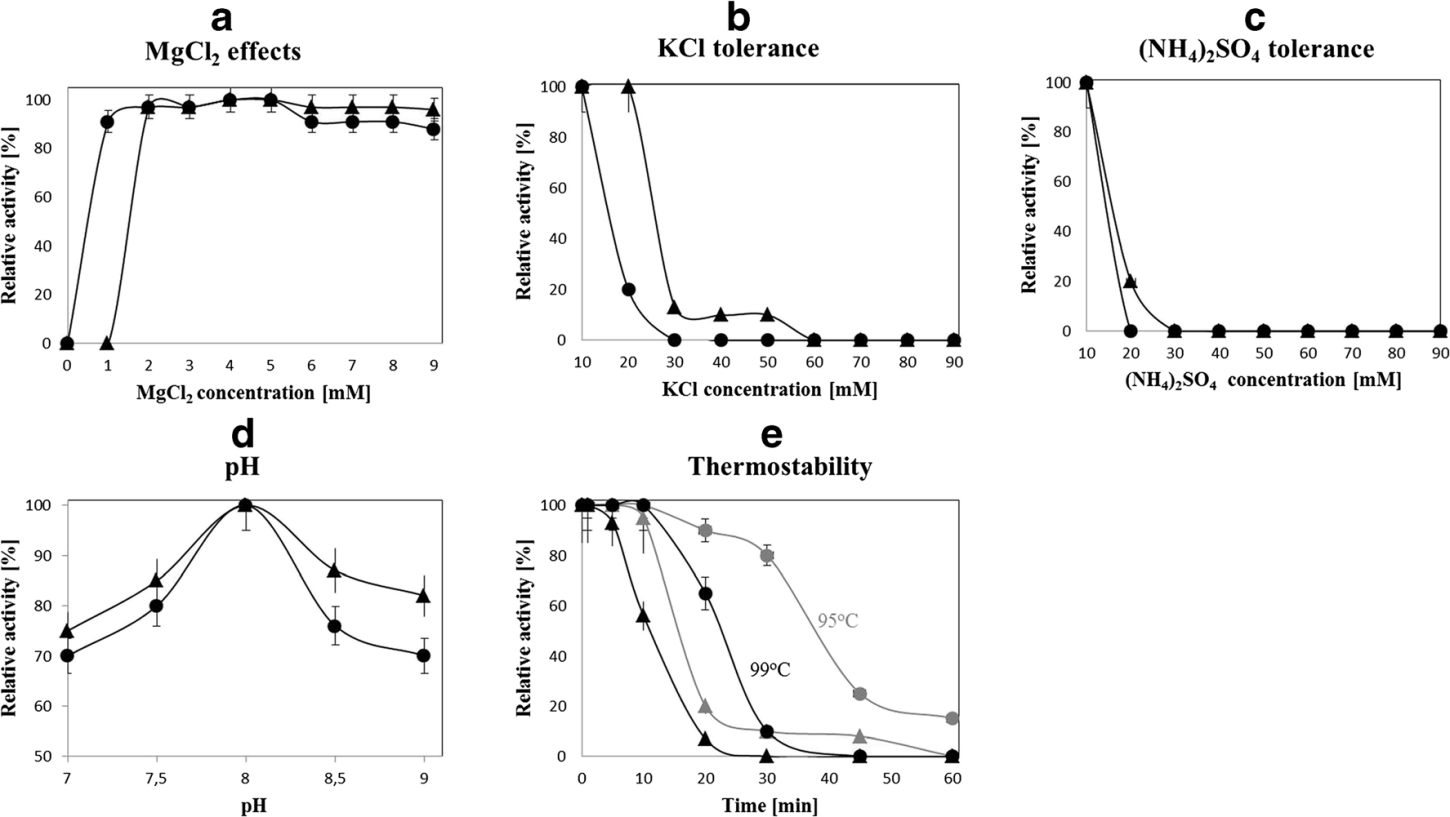
Characterization of the fusion *Pfu*DBDlig-TaqS DNA polymerase in comparison to the TaqS DNA polymerase. The effect of **a** MgCl_2_, **b** KCl, **c** (NH_4_)_2_SO_4_, **d** pH, and **e** temperature on polymerase activity: gray 95 °C; black 99 °C. Error bars for *Pfu*DBDlig*-TaqS* DNA polymerase have the end bar, for *Taq*S DNA polymerase, do not have the end bar

For characterization purposes, the polymerase activity was measured by PCR using various buffer compositions and concentrations of MgCl_2_, KCl, or (NH_4_)_2_SO_4_ and various pHs (Fig. 2).

MgCl_2_ concentration had a strong effect on the activity of *Taq*S and *Pfu*DBDlig-TaqS, which was optimal for MgCl_2_ equal 2 to 5 mM and 1 to 5 mM, respectively (Fig. 2a).

The DNA polymerase activity was completely inhibited when KCl concentrations exceeded 60 mM for *Taq*S and 30 mM for *Pfu*DBDlig-*Taq*S (Fig. 2b). *Taq*S and *Pfu*DBDlig-*Taq*S DNA polymerase activities were also strongly affected by (NH_4_)_2_SO_4_ and were completely inhibited at concentrations of over 30 and 20 mM, respectively (Fig. 2c). The fusion of *Pfu*DBDlig to the *Taq*S DNA polymerase resulted in a low tolerance to salt inhibition in PCR.

The effect of pH on the activity of *Taq*S and *Pfu*DBDlig-*Taq*S DNA polymerases was evaluated using buffers with a various pH ranging from 7 to 9. Both polymerases had the highest enzyme activity at pH 8.0 (Fig. 2d).

Based on the results presented in this study, we found that the optimal PCR buffer for the *Pfu*DBDlig-*Taq*S DNA polymerase consisted of 20 mM Tris–HCl (pH 8.0), 4 mM MgCl_2_,10 mM (NH_4_)_2_SO_4_, and 10 mM KCl.

Thermostability of *Taq*S and *Pfu*DBDlig-*Taq*S DNA polymerases was tested by measuring the decrease in their activity after preincubation at 95 or 99 °C and was found to be remarkably higher for *Pfu*DBDlig-*Taq*S. *Taq*S and *Pfu*DBDlig-*Taq*S DNA were also found to have a half-life of 10 and 23 min at 99 °C, respectively (Fig. 2e).

### PCR amplification rate and processivity

**Fig. 3:**
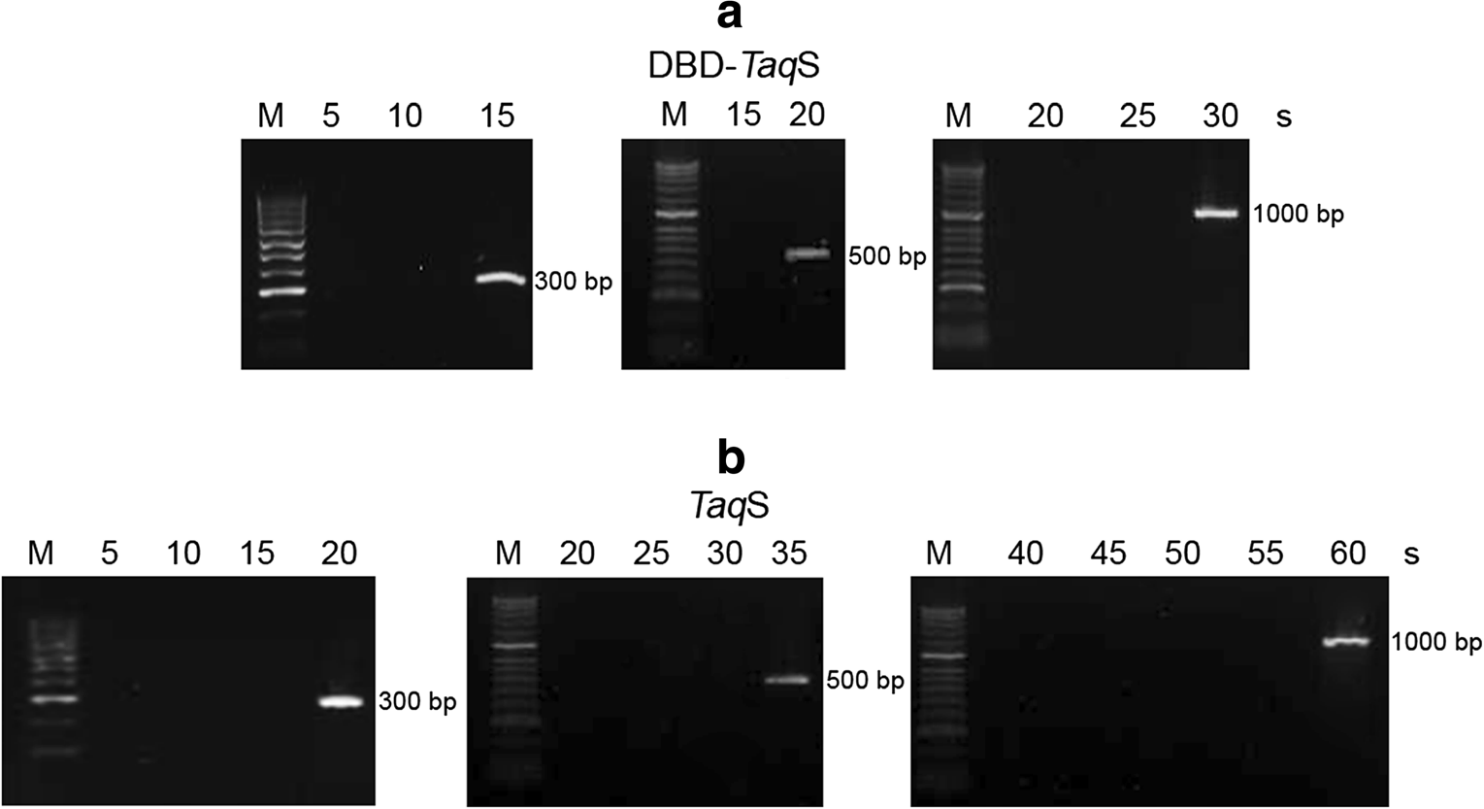
Comparison of PCR amplification rates of the fusion *Pfu*DBDlig-TaqS DNA polymerase (**a**) and the *Taq*S DNA polymerase (**b**). The elongation times used for PCR amplification are shown at the top. Lane M, DNA molecular size marker (100–1000 bp) (Blirt, Gdańsk, Poland). The amplified products were analyzed in a 1.5% agarose gel stained with ethidium bromide

The fusion *Pfu*DBDlig-*Taq*S DNA polymerase was found to replicate template strands at a faster rate than did the *Taq*S DNA polymerase (Fig. 3), namely, it replicated a 300-bp template within 15 s, a 500-bp template within 20 s, and a 1000-bp template within 30 s, while the *Taq*S DNA polymerase required 20 s for a 300-bp template, 35 s for a 500-bp template, and 1 min for a 1000-bp template. This showed that the fusion of the *Pfu*DBDlig protein with the *Taq*S DNA polymerase was two times more efficient and the amplification of DNA required less time.

**Fig. 4:**
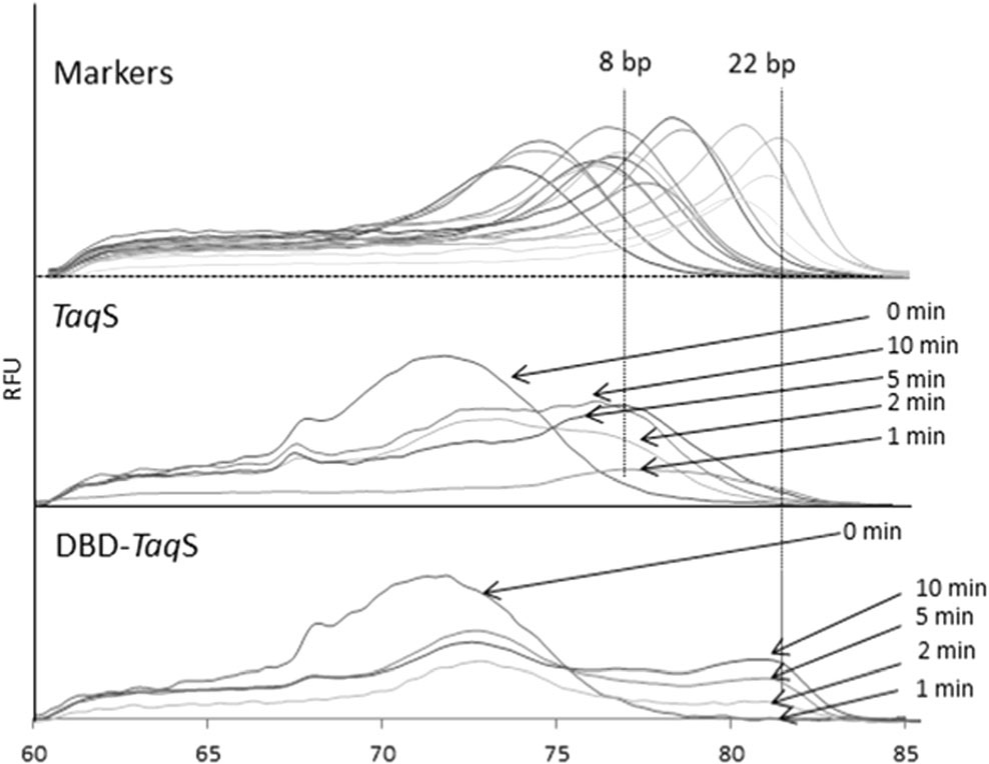
Determination of processivity by measuring the melting temperature of DNA products in the presence of a heparin trap

To determine processivity, the primer template and the polymerase were pre-incubated in the absence of Mg^2+^ and the reaction was initiated by the simultaneous addition of a metal and heparin trap. Next, processivity was determined by comparing the melting temperature profiles of the targets with the profiles of the products of a known length which served as markers in the reaction. In the case of the *Pfu*DBDlig-*Taq*S DNA polymerase, a prominent melting pick is seen resulting from the incorporation of 22 nucleotides and representing processivity (Fig. 4). The control *Taq*S DNA polymerase had a prominent melting pick which indicated lower processivity in the order of 8 nucleotides. This shows that a fusion of the *Taq*S DNA polymerase with the *Pfu*DBDlig strongly improves processivity.

### Sensitivity

**Fig. 5:**
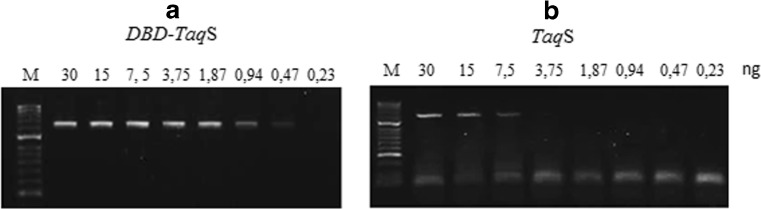
Electrophoretic separation showing the products of plasmid DNA amplification as a function of a template concentration with the use of the fusion polymerase PfuDBDlig-TaqS polymerase (**a**) and the TaqS polymerase (**b**). Lane M, marker HyperLadder II (Bioline, UK). The amplified products were analyzed in a 1.2% agarose gel stained with ethidium bromide

Sensitivity was measured in a PCR with the use of *Pfu*DBDlig-*Taq*S and *Taq*S polymerases with two-fold serial dilutions of template, and the size of the PCR product was 1264 bp. The experiment showed that the fusion polymerase was more sensitive than the reference polymerase. In the case of the fusion polymerase, it was sufficient to use 0.47 ng of plasmid DNA, while the *Taq*S polymerase required at least 7.5 ng of the plasmid. The results are presented in Fig. 5.

### Tolerance of the fusion *Pfu*DBDlig DNA polymerase to PCR inhibitors

**Fig. 6:**
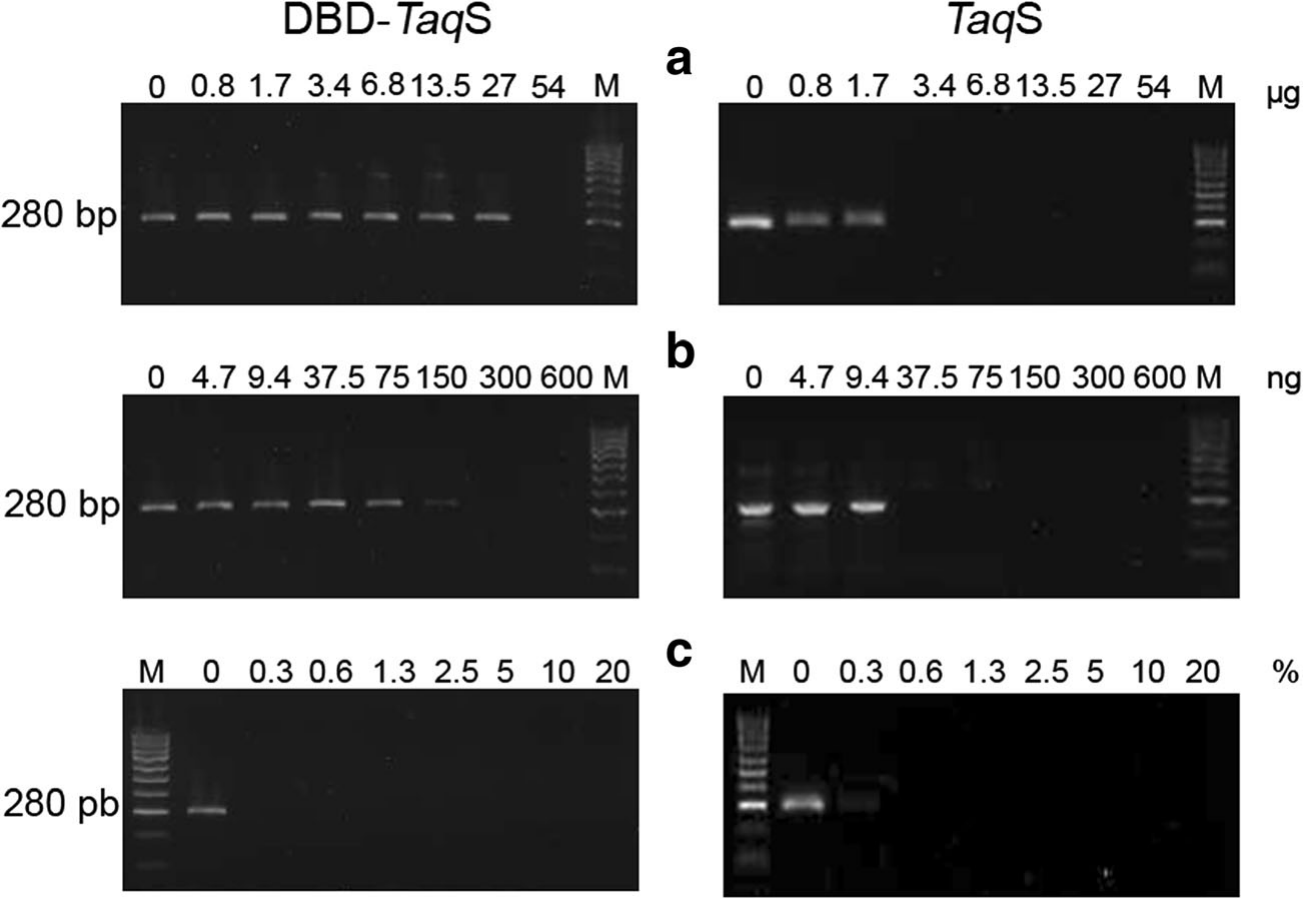
The **e**ffect of lactoferrin (**a**), heparin (**b**), and blood (**c**) inhibitors on DNA amplification using the genomic DNA of *S .aureus* as a template and primers for *S .aureus*-specific *nuc* gene detection. Control reactions were carried out without any inhibitors. Lane M, DNA standards ladder (100–1000 bp) (Blirt, Gdańsk, Poland). The amplified products were analyzed in a 2% agarose gel stained with ethidium bromide

The fusion *Pfu*DBDlig-*Taq*S enzyme and the *Taq*S DNA polymerase were PCR-tested for their resistance to serial dilutions of whole human blood, lactoferrin, and heparin, which have been reported to be PCR inhibitors in many publications (Al-Soud and Rådström 1998, 2000, 2001; Yokota et al. 1999; Kermekchiev et al. 2008). It was shown that the fusion *Pfu*DBDlig-*Taq*SDNA polymerase was significantly more resistant to lactoferrin and heparin than the *Taq*Stoffel enzyme and had no resistance to blood. The *Pfu*DBDlig-*Taq*S polymerase remained functional in the presence of 0.84–27.0 μg of lactoferrin vs. 1.68 μg for the *Taq*S enzyme (Fig. 6a) and 4.7–150 ng of heparin vs. 9.4 ng for the *Taq*S enzyme (Fig. 6b).

### Long-range PCR efficiency

**Fig. 7:**
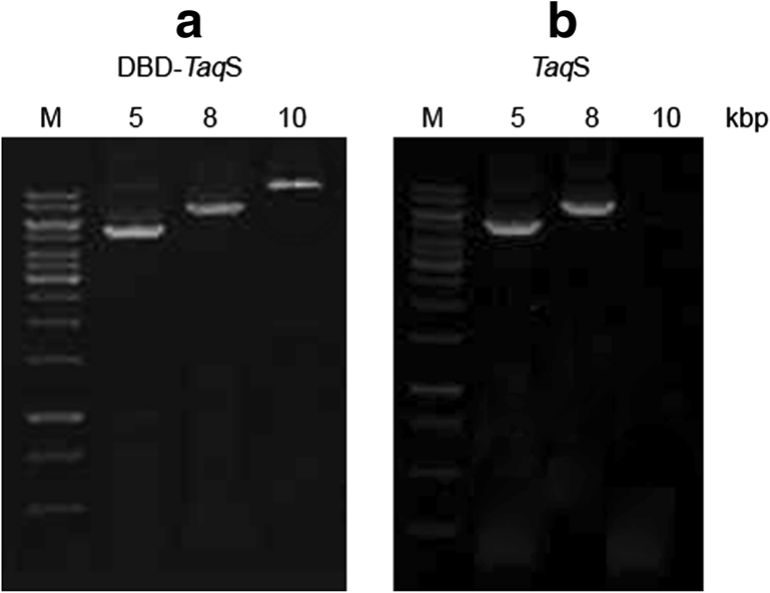
Comparison of the PCR efficiency of the *Pfu*DBDlig-*Taq*S DNA polymerase (**a**) and the *Taq*S DNA polymerase (**b**). DNA polymerases were used to amplify 5-, 8-, and 10-kbp targets from *E. coli* genomic DNA with the use of various extension times—72 °C, 1 min/kb. Lane M, GeneRuler DNA ladder SM 1331 (75–20,000 bp) (ThermoScientific, USA) The amplified products were analyzed in a 1% agarose gel stained with ethidium bromide

The long-term PCR amplification efficiency of *Pfu*DBDlig-*Taq*S was tested using DNA fragments of various sizes, including 5-, 8-, and 10-kbp DNA targets, various primer combinations and the abovementioned optimal PCR buffer. The results were compared with what was achieved with the use of the *Taq*S DNA polymerase. The PCR amplification efficiency of *Pfu*DBDlig-*Taq*S DNA polymerase using 5 and 8 kbp targets was similar to the efficiency of the *Taq*S DNA polymerase (Fig. 7). However, the fusion *Pfu*DBDlig-*Taq*S DNA polymerase amplified longer DNA fragments more efficiently than *Taq*Stoffel: 10 and 8 kbp, respectively.

### GC-rich templates

**Fig. 8:**
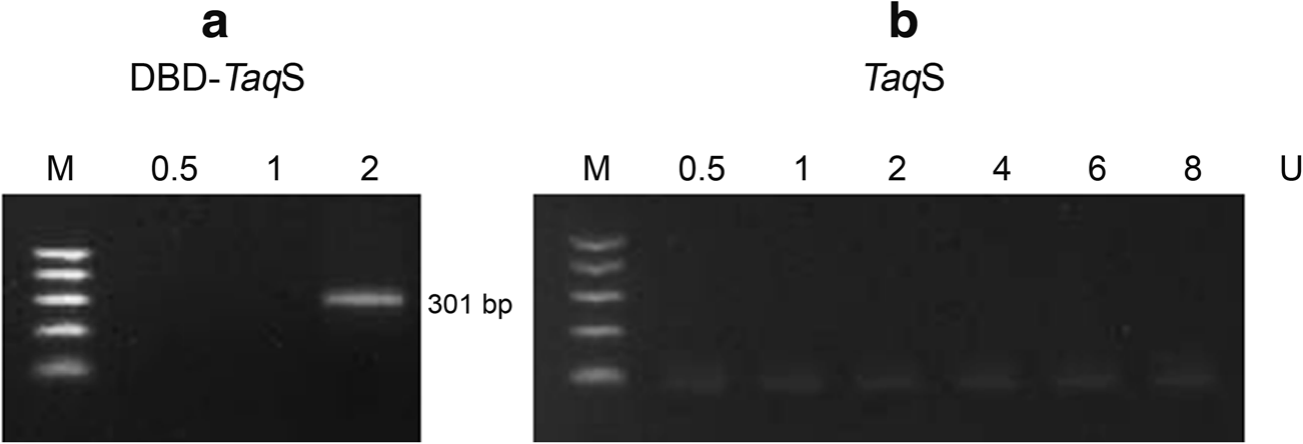
Comparison of *Pfu*DBDlig-*Taq*S DNA polymerase (**a**) and the *Taq*S DNA polymerase (**b**) PCR efficiency for templates with high content of GC. DNA polymerases were used to amplify 78% GC-rich target from *M. tuberculosis* genomic DNA for various polymerase concentrations (0.5–8 U). Lane M, DNA ladder (100–500 bp) (Blirt, Gdańsk, Poland). The amplified products were analyzed in a 2% agarose gel stained with ethidium bromide

The potential of the fusion polymerase to amplify products with a high GC content was evaluated with the use of *Pfu*DBDlig-*Taq*S and *TaqS* under the optimal conditions*.* A genomic DNA fragment of *Mycobacterium tuberculosis* with a GC content of 78% was amplified with the use of various polymerase concentrations: 0.5–8 U. Figure 8 presents the results of the experiment. It was shown that the fusion polymerase *Pfu*DBDlig-*Taq*S had the ability to efficiently amplify a product with a high GC content with the use of only 2 U of polymerase, while the reference *TaqS* polymerase was shown not to work even where 8 U of polymerase were used.

## Discussion

Currently, the PCR method is widely used in diagnostics, molecular biology, and genetic engineering. PCR effectiveness, or in other words amplification yield and accuracy, depends on the type of polymerase and PCR conditions. Many problems are encountered during the PCR amplification of so-called difficult templates. These primarily include large products, products with a high content of high-melting GC pairs or templates of clinical or environmental samples containing numerous DNA polymerase inhibitors. One of the solutions could be the use of a fusion polymerase with an additional DNA-binding domain. Research has indicated (Oscorbin et al. 2015) that the addition of a DNA-binding domain of DNA ligase from *Pyrococcus abyssi* to a PCR mixture has a positive effect on the activity of the *Taq* DNA polymerase in the presence of difficult templates. The literature indicates primarily an improvement in the PCR efficiency for long products (4 and 8 kbp) and a considerable reduction in the inhibitory effect of heparin in the reaction mixture.

Our studies have confirmed the results obtained by Oscorbin et al. It was shown that the fusion of a DBD of ligase (from *Pyrococcus furiosus*) to DNA polymerases improves basic enzyme properties such as thermostability, processivity, or reaction sensitivity. Polymerases containing a DBD of ligase are an ideal solution for PCR amplification of difficult templates.

The half-life of the fusion *Pfu*DBDlig-*Taq*S polymerase doubled as compared to the reference *TaqS*, from 10 to 23 min at 99 °C, which predicts that the fusion polymerase will have a greater ability to amplify products with a high GC content. The *Pfu*DBDlig-*Taq*S polymerase is able to efficiently amplify products with a GC content of up to 78%, while *TaqS*, which does not contain an additional domain, fails with targets with such a high GC content.

A two-fold increase in the amplification speed (from 16 to 33 nt/s) and an almost three-fold improvement of the processivity (from 8 to 22 nt) were found to increase the fusion polymerase’s ability to amplify large products by 25% (from 8 to 10 kb). Based on the results of the studies, it appears that the *Taq*Stoffel polymerase is able to amplify targets with a size of 8 kb, while the fusion *Pfu*DBDlig-*Taq*S polymerase, under the same conditions, of up to 10 kb .

Another example of problematic amplification is a PCR carried out in the presence of inhibitors. The fusion polymerase appeared again to have better properties than the reference *Taq*S. The *Pfu*DBDlig-*Taq*S polymerase tolerates, in a reaction mixture, an almost 16-fold higher concentration of lactoferrin and an eightfold higher concentration of heparin which are both primary inhibitors found in clinical and environmental samples (Al-Soud and Rådström 1998, 2001; Yokota et al. 1999). Such an improvement may be due to a 16-fold increase in sensitivity of the fusion polymerase which therefore has a higher affinity for the template. It seems that the template is picked up more selectively from the mixture in the presence of a binding domain and the PCR is more efficient even when inhibitors are present.

The fusion *Pfu*DBDlig-*Taq*S DNA polymerase consisting of a *Taq*Stoffel polymerase and the DNA-binding domain of a ligase obtained from *Pyrococcus furiosus* exhibits a higher extension rate, higher processivity, thermostability, sensitivity, and a higher tolerance to some PCR inhibitors and is able to amplify templates with a higher GC content and longer PCR products than the *Taq*Stoffel DNA polymerase. For this reason, it may be a better solution to use the *Pfu*DBDlig-*Taq*S DNA polymerase instead of the *Taq* DNA polymerase in many PCR applications with difficult targets.
